# Trajectories of trace element accumulation in seagrass (*Posidonia oceanica*) over a decade reveal the footprint of fish farming

**DOI:** 10.1007/s11356-024-32910-0

**Published:** 2024-03-26

**Authors:** Victoria Litsi-Mizan, Ioanna Kalantzi, Manolis Tsapakis, Spiros A. Pergantis, Ioannis Karakassis, Eugenia T. Apostolaki

**Affiliations:** 1 Biology Department, University of Crete, Voutes University Campus, P.O. Box 2208, 70013 Heraklion, Crete Greece; 2 Institute of Oceanography, Hellenic Centre for Marine Research, P.O. Box 2214, 71003 Heraklion, Crete Greece; 3 Department of Chemistry, Environmental Chemical Processes Laboratory, University of Crete, Voutes University Campus, P.O. Box 2208 70013, Heraklion, Crete Greece

**Keywords:** Aquaculture, Reconstruction, Metals, Macrophytes, Lepidochronology, Bioindicator, Pollution, Mediterranean Sea

## Abstract

To evaluate the effect of trace element (TE) release from fish farms on seagrass *Posidonia oceanica*, we compared TE concentrations (As, Cd, Co, Cu, Mn, Mo, Ni, Pb, V, Zn) in shoots near fish cages (Station ‘Cage’) with those away from them (Station ‘Control’) in two fish farm facilities (Site 1 and Site 2, North Aegean Sea, Greece). We assessed the present (i.e., 2021, year of sampling) and past (reconstructed period 2012–2020) accumulation of TEs using the living compartments (leaf blades, sheaths, rhizomes, roots, epiphytes) and the dead sheaths, respectively. We also assessed possible seagrass degradation by reconstructing past rhizome production. *P*. *oceanica rhizome *production at the ‘Cage’ stations was up to 50% lower than at the ‘Control’ stations. Most TE concentrations were higher at ‘Cage’ stations, but the differences often depended on the seagrass living compartment. Significant differentiation between ‘Cage’ and ‘Control’ stations was observed based on the TE concentrations of the dead sheaths during 2012–2020. The contamination level at the ‘Cage’ stations was mostly moderate in Site 1 and low in Site 2, during the reconstructed period, while an increasing contamination trend was found for certain potential phytotoxic TEs (As, Cu, Cd, Mo, V). Our results emphasize the need for the aquaculture industry to work towards a more ecologically aware approach.

**Supplementary Information:**

The online version contains supplementary material available at 10.1007/s11356-024-32910-0.

## Introduction

Aquaculture has been a rapidly growing industry since the 1990s, with an annual growth rate of about 7% worldwide (FAO 2022). Despite the undeniable value of the aquaculture sector in meeting growing food demands and supporting local economies, its expansion raises concerns due to the often adverse effects it imposes on the surrounding marine ecosystems and the subsequent alterations in biodiversity and ecosystem functionality (Ottinger et al. 2016; Weitzman et al. 2019; Tičina et al. 2020). The effects are particularly detrimental to seagrass ecosystems and include alterations of morphological, structural, physiological and biochemical traits resulting in extensive seagrass decline (Pergent-Martini et al. 2006; Holmer et al. 2008; Boudouresque et al. 2020; Howarth et al. 2022). The iconic, endemic seagrass species of the Mediterranean Sea, *Posidonia oceanica* (L.) Delile, often found in the proximity of fish farm facilities, shows decreased growth or even mortality (Duarte et al. 2006; Díaz-Almela et al. 2008; Apostolaki et al. 2009; Kletou et al. 2018) that may persist even after the cessation of fish farming (Delgado et al. 1999).

Aquaculture impacts primarily originate from the increased release of dissolved and particulate matter and chemicals (e.g., metals, feed additives, antibacterials) in the water column and their accumulation in the underlying sediment. Of particular concern is the release of trace elements (TEs), many of which are important components of aquaculture feeds (Kalantzi et al. 2013). Additional TE sources include wastes, such as uneaten feed pellets and fish excreta, along with antifouling paints (Grigorakis and Rigos 2011; Kalantzi et al. 2013; Farmaki et al. 2014). Despite being usually present in relatively small quantities, TEs are considered dangerous contaminants due to their lack of biodegradability and their high bioaccumulation capacity (Lewis and Devereux 2009). When TEs enter the marine environment, they partition between the aqueous and solid phases and, depending on the prevailing hydrodynamic, environmental and physicochemical conditions, they become bioavailable, taken up by various organisms and transferred throughout the food chain (Eggleton and Thomas 2004; Zhang et al. 2019; Marengo et al. 2023). While some of these elements serve as essential micronutrients for seagrass, they can become toxic when certain concentrations are exceeded, compromising some of their key functional traits (e.g., Cu, Zn; Ralph and Burchett 1998; Lin et al. 2016; Llagostera et al. 2016). Other TEs (e.g., Cd, Pb), despite being nonessential for seagrasses, can be retained by their tissues and lead to toxic effects when present at high concentrations (see Lewis and Devereux 2009 for a review). TE accumulation by seagrass can incite phytotoxic episodes, subsequently undermining their structural integrity, photosynthetic capacity, biochemical mechanisms and growth (see Bonanno and Orlando-Bonaca 2018; Li et al. 2023 for reviews). Examples of TE toxicity in seagrass include decrease of growth and increase in leaf necrosis in *Cymodocea nodosa* (Llagostera et al. 2016), photoinhibition in *Zostera muelleri* (Buapet et al. 2019) or induced biochemical changes in *Zostera japonica* (Lin et al. 2016).While some seagrass species, like *P*. *oceanica* have occasionally shown high tolerance to TEs, the precise toxicity threshold values often remain elusive (Bonanno and Orlando-Bonaca 2017).

To comprehensively evaluate the prolonged consequences of persistent TE release arising from direct anthropogenic activities, such as fish farming, it is crucial to examine their trajectory over the long term. In this regard, *P*. *oceanica* is a valuable bioindicator which not only takes up elements from both the water column and the sediment, but also facilitates their transfer to higher trophic levels through grazing reflecting the bioavailability of elements in the environment (Malea et al. 2019a). Different tissues of *P*. *oceanica*, such as leaves, rhizomes and roots, have been successfully utilized to assess the accumulation patterns of different TEs (Sanz-Lázaro et al. 2012; Malea et al. 2019a), frequently associated with direct anthropogenic activities (Lafabrie et al. 2007b, a; Roca et al. 2017).

Except from its ability to detect recent contamination signals, *P*. *oceanica* can integrate and record environmental changes over extended periods. This capacity is attributed to the unique characteristic of *P*. *oceanica* sheaths to remain attached to the rhizome after the leaf blades are shed which provide the opportunity to investigate past TE concentrations by applying a retrospective technique (lepidochronology; Pergent 1990). Even though efforts have been directed towards improving the utility of *P*. *oceanica* as a bioindicator of past TE contamination (Malea et al. 2019b), the application of lepidochronology to investigate the trajectory of TEs in seagrass remains limited, as evidenced by its use in a few studies (Gosselin et al. 2006; Lafabrie et al. 2007a; Tovar-Sánchez et al. 2010; Malea et al. 2019b). In addition, the association between TE trajectories and direct human activities has been infrequent (Pergent et al. 1999; Ancora et al. 2004; Copat et al. 2012). Surprisingly, despite the rapid growth of the fish farm industry in the Mediterranean basin, particularly in finfish aquaculture, there has been a lack of long-term assessments of TE accumulation patterns associated with that activity (Pergent et al. 1999). This knowledge gap underscores the need for a comprehensive investigation of the persistent impact of fish farming on *P*. *oceanica* and the surrounding ecosystem.

In the present study, we assessed the trajectory of TE accumulation in seagrass meadows adjacent to fish farm activities over a decade (2012–2021). To achieve this, we measured TE concentrations in living compartments (i.e., leaf blades, sheaths, rhizomes, roots and epiphytes), as well as in the dead sheaths of *P*. *oceanica* shoots from the vicinity of two fish farm facilities in the North Aegean Sea (Greece). Their concentrations were compared to the corresponding concentrations in *P. oceanica *shoots found away from the fish farm facilities. We also investigated the seagrass rhizome production during the reconstructed period to detect signs of meadow degradation attributed to fish farm activities. Finally, we evaluated the TE contamination levels in *P*. *oceanica*, as well as the contamination temporal trend by calculating the contamination factor (CF) of the dead sheaths near the fish farm cages over the investigated period.

## Materials and methods

### Study area and sample collection

**Fig. 1:**
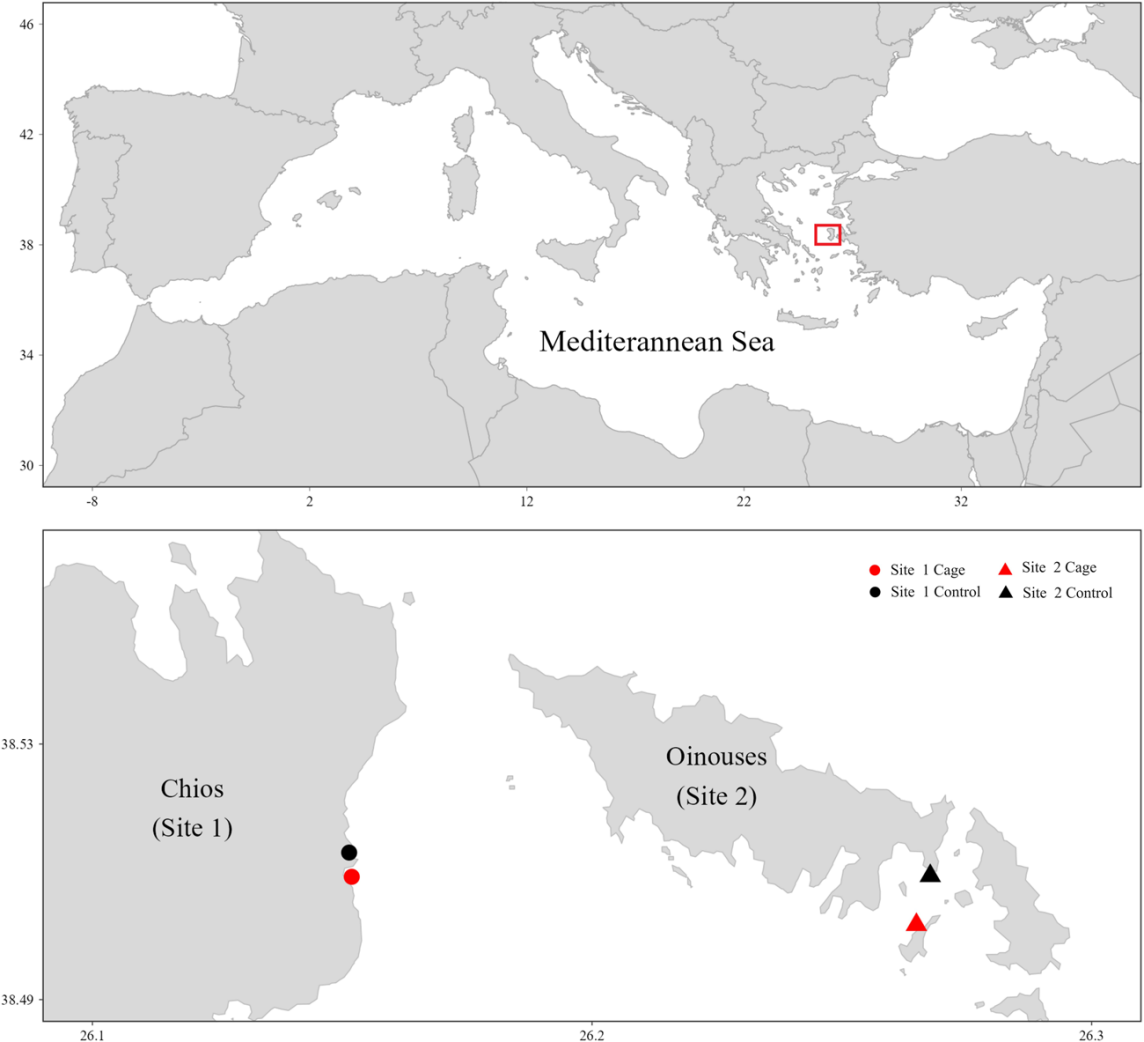
Map of the study area with sites (Site 1, Site 2) and stations (Cage, Control) situated in the North Aegean Sea, Greece

The study area was in the North Aegean Sea, Greece. Two sites were selected: Chios (hereafter called ‘Site 1’) and Oinouses (hereafter called ‘Site 2’) (Fig. 1). Sampling was conducted in September 2021. The fish farms have been operating since 1992 and produce mainly seabass (*Sparus aurata*) and seabream (*Disentrarchus labra*x) between 10 and 500 g in size. Both fish farms follow similar management approaches and therefore no major differences in the TE sources (e.g., feeds and antifouling paints used), should be expected.

At each site, two stations were selected based on the presence of *P*. *oceanica* meadows. One station (hereafter called ‘Cage’) was selected as close to the fish cages as possible, which was 60 m and 65 m away from the cages in Site 1 and Site 2, respectively. The other station (hereafter called ‘Control’) was selected as a reference station and was outside the zone affected by the fish farm activities (Holmer et al. 2008) at a distance of 400 m from Site 1 and 600 m from site 2, depending on *P*. *oceanica* meadows presence (Fig. 1). A minimum safety distance of 400 m has been determined as an adequate distance for seagrass meadows, after which the effects of aquaculture activities are minimized (Holmer et al. 2008). This distance was established through a comprehensive synthesis of data from fish farms across the Mediterranean Sea, encompassing various parameters of the water column, sediment, benthic fauna and seagrass. In addition, we applied the MaLE method (Malea et al. 2019b) to affirm the suitability of the selected ‘Control’ sites as reference stations for TE contamination assessment. The method was applied only for the TEs for which it has been developed and coincide with the ones used in the present study (i.e., Co, Cu, Ni, Pb). Based on the application of the MaLE method, the selected ‘Control’ stations were characterized by ‘low’ contamination levels (CF < 1), classifying them as ‘non-polluted’ in all cases (Fig. S1).

The water depth was 3–5 m. At each station, fifteen orthotropic shoots were randomly collected by SCUBA divers.

### Laboratory and analytical procedures

The *P*. *oceanica* shoots were brought to the laboratory and pooled together to form three replicates, each consisting of five shoots. The epiphytes were gently removed from the leaves, using a plastic blade. Each shoot was separated into its living compartments, which included the epiphytes, the leaf blades and sheaths, the rhizomes and the roots.

The rhizome per shoot was used to apply the reconstructive technique of lepidochronology (Pergent 1990). This method is based on the characteristic of *P*. *oceanica* sheaths to remain attached to the rhizome after the leaf blades are shed. The remaining sheaths (dead sheaths) exhibit annual cyclic variations in their thickness. Starting from the rhizome apex and downwards, one lepidochronological year was determined between two successive minima in sheath thickness. The thickness of the sheaths was measured in their central part using an electronic calliper (Guidetti et al. 2000; Stipcich et al. 2022; Litsi‐Mizan et al. 2023). The consecutive rhizome segments (i.e., corresponding to different years) were cut and dried in a lyophilizer to estimate seagrass rhizome production (mg DW shoot^−1^ year^−1^) as the dry weight of each rhizome segment corresponding to each reconstructed year. In the present study, rhizome production always refers to orthotropic rhizomes. The rhizome growth is often considered incomplete in the two most recent years (Peirano 2002), and, therefore, those years (i.e., 2020 and 2021) were excluded from the subsequent analysis of rhizome production data.

The living compartments and the dead sheaths of each year were also lyophilized and milled to a fine powder using a metal-free agate mill to determine the concentration of each TE (μg g^−1^ DW). The concentrations were measured following a modified method by USEPA Method 3052 (1996). Each sample (~ 0.1 g) was digested in acid-cleaned high-pressure Teflon vessels with 5 ml of concentrated HNO_3_^−^ and 2 ml of concentrated H_2_0_2_. The vessels were sealed and pre-heated using a sand bath of 125 °C for approximately 1 h. All the tubes were then transferred into a closed microwave system (Multiwave 3000, Anton Paar, Austria), where they were digested for approximately 75 min. After digestion, samples were diluted with deionized water, transferred to 50 ml volumetric flasks and stored at4 °C until further analysis. Elemental concentrations were measured in the sample digests by inductively coupled mass spectrometry (ICP-MS NexION350, PerkinElmer, Shelton, CT, US) according to USEPA Method 6020A (2007). An internal standard of indium and bismuth was added to each sample. A reference standard was also measured every 10 samples.

The accuracy of the measurements was assured by analysing one blank sample and one certified reference material (CRMs) every six biological samples. The CRMs that were used were the aquatic plant *Lagarosiphon major* (BCR-060) and the mussel tissue (BCR-668) certified by the Joint Research Center (JRC), and the fish protein (DORM-4) and the non-defatted lobster hepatopancreas (LUTS1) certified by the National Research Council of Canada (NRC-CNRC), with respective average recoveries of 87 ± 15.1%, 103 ± 4.6%, 99 ± 10.8% and 106 ± 10.3%. Ten TEs (As, Cd, Cu, Co, Mn, Mo, Ni, Pb, V, Zn) were chosen for further analysis based on the accuracy of their measurements and their relevance to fish farm activities (Emenike et al. 2022).

To calculate the limits of detection (LOD) for each TE, the standard deviation of the blanks was multiplied by three. The LODs for each TE were on average: 0.05 (As), 0.005 (Cd), 0.29 (Cu), 0.04 (Co), 0.42 (Mn), 0.01 (Mo), 0.55 (Ni), 0.04 (Pb), 0.12 (V) and 7.21 (Zn) μg g^−1^ DW. For each sample and TE, the three replicates were checked for precision based on their relative standard deviation (RSD) and those that were considered outliers (RSD > 90%) were removed from subsequent analysis. When more than 50% of the samples had concentrations that exceeded the LOD, the concentrations that were below the LOD were replaced by LOD/2 (USEPA 1991).

### Numerical procedures

Data analyses were performed for each site separately in order to examine the variability of data between stations within each site and avoid potential obscuration of the results by the differences between sites owing to factors other than the proximity to fish cages (e.g., geomorphology).

All statistical analyses were concentrated on comparing TE concentrations between the ‘Cage’ and ‘Control’ stations within the same lepidochronological year. This approach aimed to counteract misinterpretations of the results that could derive from the potential overestimation of TE concentrations due to the loss of sheath biomass during the decay process (Malea et al. 2019b). Considering that sheath degradation can be assumed to be similar within the same lepidochronological year (since the sheaths are at the same physiological state), especially at comparable depths (‘Control’ and ‘Cage’ stations are at depths of 3–5 m), this approach helps account for any variations.

Analysis of covariance (ANCOVA) was performed in each site to examine differences in seagrass rhizome production and TE concentrations in the dead sheaths between the two stations during the reconstructed period. For this purpose, ‘Station’ was used as a categorical independent variable and ‘Year’ was used as a continuous covariate. When the interaction term ‘Station × Year’ was significant (*p* < 0.05), it suggested different temporal trends between the two stations. In these cases, separate linear regression analyses were applied to estimate the different temporal trends between the two stations. The living sheaths were not included in this analysis as the lepidochronological year might have not been complete at the time of sampling (Peirano 2002) and could bias the results.

In each site, differences in TEs concentrations between stations and living compartments were examined using a two-way analysis of variance (ANOVA), with ‘Station’ and ‘Compartment’ as fixed factors. In cases where a significant interaction was found (Station × Compartment), this meant that differences in TE concentrations between stations depended on the compartment. In these cases, separate one-way ANOVAs were performed for each compartment to identify the specific compartments in which the stations exhibited significant differences.

A principal component analysis (PCA) was used to determine the patterns of variation in TE concentrations in the dead sheaths across ‘Cage’ and ‘Control’ stations at each site during the reconstructed period. The PCA utilized a Euclidean distance matrix of concentrations per TE and year. To assess the statistical significance of differences between ‘Cage’ and ‘Control’ stations at each site, a permutational analysis of variance (PERMANOVA, number of permutations = 999) was used. Prior to analysis, all data were standardized using z-scores.

The contamination factor (CF) per year was calculated as follows:

$$\mathrm{Contamination\;factor}\;({\text{CF}}): {{\text{C}}}_{{\text{cage}}}/{{\text{C}}}_{{\text{control}}},$$

where C_cage_ and C_control_ were the mean concentrations of each TE in the dead sheaths at stations ‘Cage’ and ‘Control’, respectively. The levels of contamination were classified as low (CF < 1), moderate (1 ≤ CF < 3), considerable (3 ≤ CF < 6) or very high (CF > 6), according to Hakanson 1980.

The temporal trends of CF were determined by linear regressions for each TE individually.

In each parametric test used above, the normality of the data was evaluated using Q-Q plots and/or Shapiro–Wilk test (Shapiro and Wilk 1965) and the homogeneity of variance among groups via Levene’s test (Levene 1960). When these assumptions were not met, data were transformed using either a base 10 logarithm or a square root.

All statistical analyses were performed in R version 4.2.2 (R Core Team 2021).

## Results

**Fig. 2:**
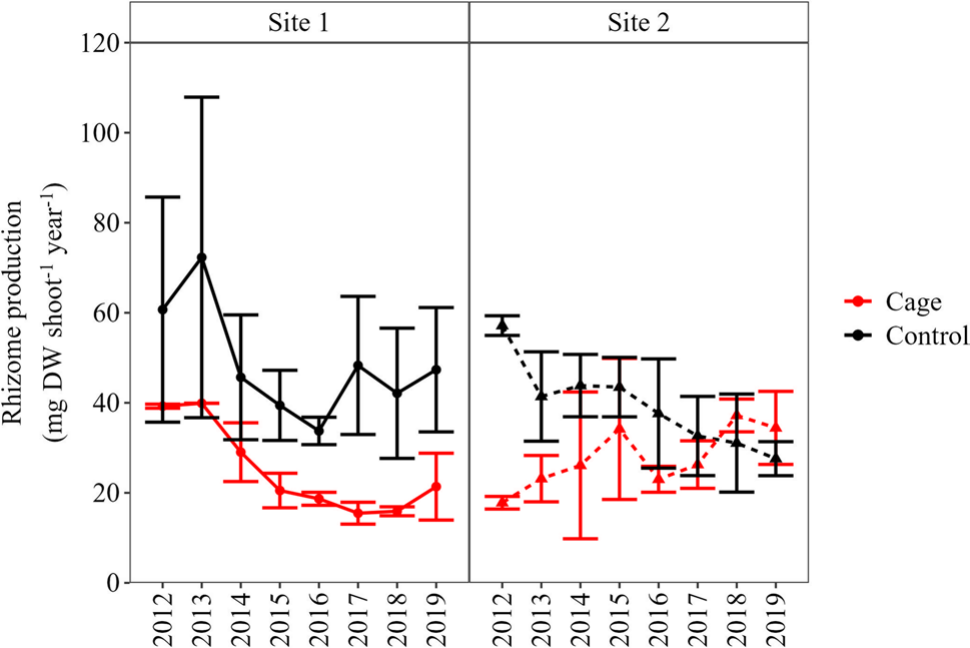
Mean (± SD) *P. oceanica* rhizome production (mg DW shoot^−1^ year^−1^) in each site and station during the period 2012–2019

Rhizome production differed between stations in both sites (Table 1), being 2 times lower at the ‘Cage’ compared to the ‘Control’ station in Site 1 (24 ± 11 and 47 ± 24 mg DW shoot^−1^ year^−1^, respectively) and 1.3 times lower at the ‘Cage’ compared to the ‘Control’ station in Site 2 (29 ± 13 and 39 ± 15 mg DW shoot^−1^ year^−1^, respectively). During the reconstructed period, the trend of rhizome production did not differ between stations in Site 1 (ANCOVA, *p* > 0.05), while it followed different trends in Site 2, remaining stable at the ‘Cage’ station (linear regression, *p* > 0.05), but decreasing at the ‘Control’ station (linear regression, R^2^ = 28%, *p* < 0.01) (Table 1, Fig. 2).

**Table 1:** Results of ANCOVA analyses performed on rhizome production and TE concentrations of the dead sheaths at two sites and stations, during the period 2012–2020

		Site 1				Site 2		
	Source of Variability	df	MS	F	*p* value	MS	F	*p* value
Rhizome production	Year	1	1639	5.3	< 0.05	344	2.1	NS
	Station	1	6020	19.6	< 0.001	1211	7.3	< 0.01
	Station × Year	1	33	0.11	NS	1766	10.8	< 0.01
As	Year	1	175	108	< 0.05	238	28.3	< 0.001
	Station	1	15.8	9.8	< 0.001	63.9	7.6	< 0.001
	Station × Year	1	38.1	23.7	< 0.001	15.7	1.9	NS
Cd	Year	1	0.07	10.1	< 0.01	0.009	1.14	NS
	Station	1	1.1	166	< 0.001	0.07	8.6	< 0.01
	Station × Year	1	0.05	7.2	< 0.05	0.05	6.01	< 0.05
Co	Year	1	0.71	27.2	< 0.001	0.49	14.3	*
	Station	1	0.43	16.2	< 0.001	0.11	3.3	ΝS
	Station × Year	1	0.0002	0.008	NS	0.03	0.9	NS
Cu	Year	1	0.38	27.2	< 0.001	0.03	1.9	NS
	Station	1	0.89	63.8	< 0.001	0.39	21.9	< 0.001
	Station × Year	1	0.04	2.7	NS	0.06	3.5	NS
Mn	Year	1	0.01	2.46	NS	198	3.32	NS
	Station	1	0.69	16.92	< 0.001	575	9.62	< 0.001
	Station × Year	1	0.13	3.29	NS	5.68	0.09	NS
Mo	Year	1	0.62	44.96	< 0.001	7.64	26.9	< 0.001
	Station	1	0.76	55.18	< 0.001	2.31	8.12	< 0.001
	Station × Year	1	0.13	9.17	< 0.001	0.81	2.88	NS
Ni	Year	1	0.44	20.3	< 0.001	3.34	1.3	NS
	Station	1	0.26	11.92	< 0.01	0.76	0.29	NS
	Station × Year	1	0.06	2.87	NS	17.9	6.9	< 0.05
Pb	Year	1	1.9	112	< 0.001	24.2	35.3	< 0.001
	Station	1	0.28	16.26	< 0.001	3.83	5.59	< 0.05
	Station × Year	1	0.03	1.62	NS	0.03	0.04	NS
V	Year	1	361	54.8	< 0.001	0.61	22.34	< 0.001
	Station	1	85.2	12.9	< 0.001	0.41	14.92	< 0.001
	Station × Year	1	4.4	0.7	NS	0.006	0.23	NS
Zn	Year	1	289	12.2	< 0.01	59.2	1.33	NS
	Station	1	723	30.5	< 0.001	239	5.39	< 0.001
	Station × Year	1	25.9	1.09	NS	51.33	1.16	NS

*NS* non-significant

**Fig. 3:**
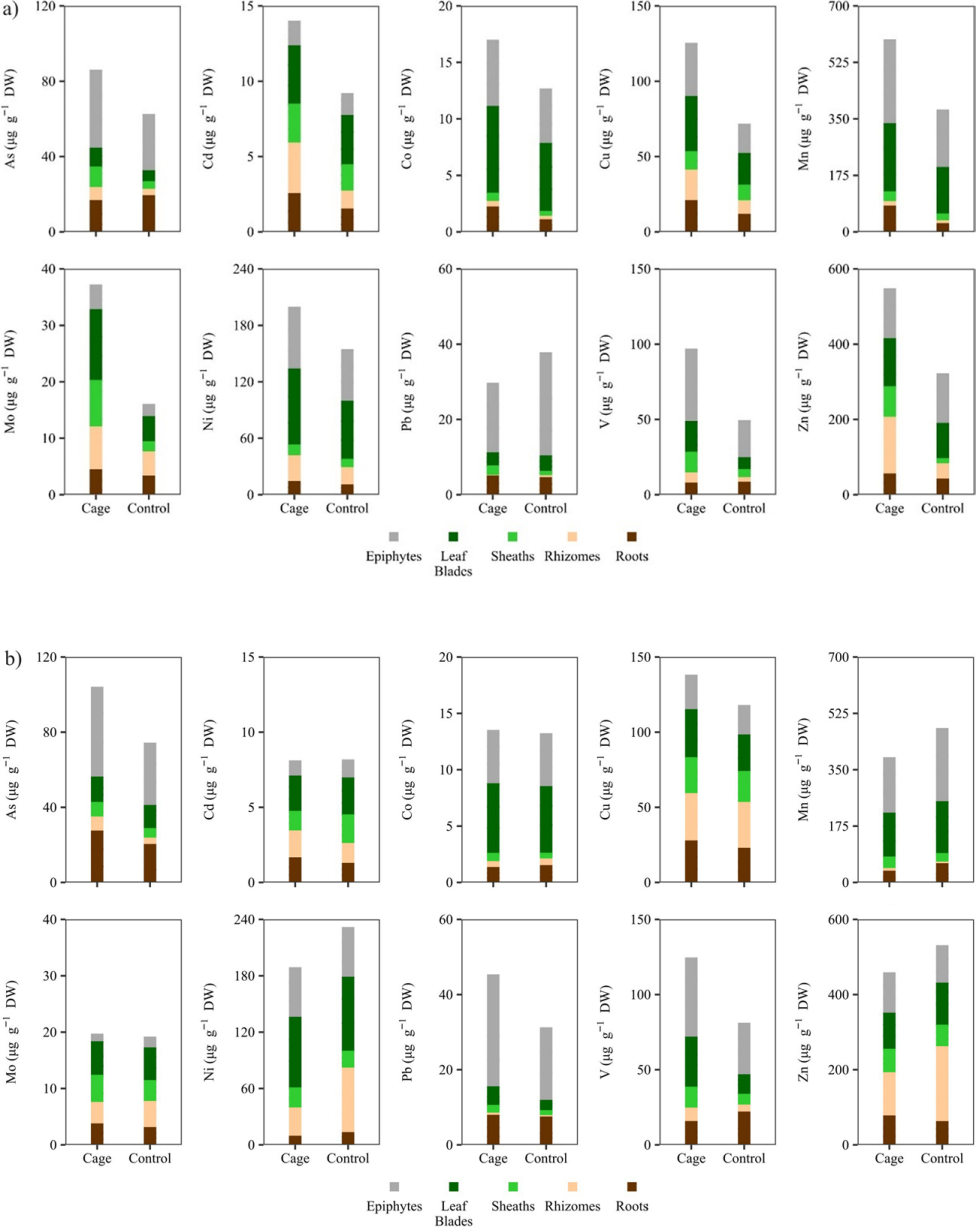
Cumulative TE concentrations (μg g^−1^ DW) at each station and living *P*. *oceanic*a compartment in 2021 in **a **Site 1 and **b **Site 2

The mean (± SD) TE concentrations (μg g^−1^ DW) for each living compartment in the two sites and stations are presented in Table S1. For most TEs, concentrations in the epiphytes ranged higher among all compartments, followed by the concentrations measured in the leaf blades (Table S1, Fig. 3). Station had a significant effect on the TE concentrations, for Co, Cu, Mn, Mo and Ni in Site 1 and for Cu in Site 2, irrespective of seagrass compartment (Table 2). However, the effect of ‘Station’ often depended on the seagrass compartment (e.g., As, Cd, Pb, V and Zn) leading to higher concentrations at the ‘Cage’ stations in particular compartments, such as epiphytes, leaf blades, sheaths or rhizomes (ANOVA, *p* < 0.05) (Table 2, Fig. 3).

**Table 2:** Results of two-way ANOVA between stations and living compartments in each site

	Source of variability	Site 1				Site 2		
		df	MS	F	*p* value	MS	F	*p* value
As	Station	1	18.3	15.7	< 0.001	0.07	2.2	NS
	Compartment	4	424	91.1	< 0.001	1.1	33.6	< 0.001
	Station x Compartment	4	17.5	3.8	< 0.05	0.05	1.5	NS
Cd	Station	1	0.8	36.0	< 0.001	0.0001	0.03	NS
	Compartment	4	0.4	18.4	< 0.001	0.17	35.5	< 0.001
	Station x Compartment	4	0.09	4.4	< 0.05	0.03	7.23	< 0.001
Co	Station	1	0.26	25.3	< 0.001	0.006	1.2	ΝS
	Compartment	4	1.92	184	< 0.001	1.5	283	< 0.001
	Station x Compartment	4	0.02	1.5	NS	0.02	3.9	NS
Cu	Station	1	126	44.6	< 0.001	13.6	8.6	< 0.01
	Compartment	4	33.8	11.9	< 0.001	15.1	9.5	< 0.001
	Station x Compartment	4	2.28	0.81	NS	1.0	0.6	NS
Mn	Station	1	0.36	23.6	< 0.001	274	3.4	NS
	Compartment	4	1.92	109	< 0.001	4602	56.9	< 0.001
	Station x Compartment	4	0.02	2.1	NS	110	1.4	NS
Mo	Station	1	1.04	30.2	< 0.001	0.004	0.08	NS
	Compartment	4	0.21	6.2	< 0.01	0.41	8.05	< 0.001
	Station x Compartment	4	0.08	2.3	NS	0.02	0.34	NS
Ni	Station	1	67.9	13.6	< 0.01	61	8.5	< 0.01
	Compartment	4	551	110	< 0.001	500	69.5	< 0.001
	Station x Compartment	4	7.4	1.48	NS	48.5	6.7	< 0.01
Pb	Station	1	0.0009	0.05	NS	0.24	7.3	< 0.05
	Compartment	4	2.02	123	< 0.001	2.04	62.5	< 0.001
	Station x Compartment	4	0.13	7.7	< 0.001	0.02	0.5	NS
V	Station	1	95.3	59.6	< 0.001	62.9	9.2	< 0.01
	Compartment	4	99.6	62.3	< 0.001	137	20.3	< 0.001
	Station x Compartment	4	11.2	7.0	< 0.01	19.9	2.9	< 0.05
Zn	Station	1	1698	31.8	< 0.001	0.003	0.50	NS
	Compartment	4	1055	19.8	< 0.001	0.19	27.1	< 0.001
	Station x Compartment	4	329	6.2	< 0.01	0.02	3.5	< 0.05

*NS* non-significant

**Fig. 4:**
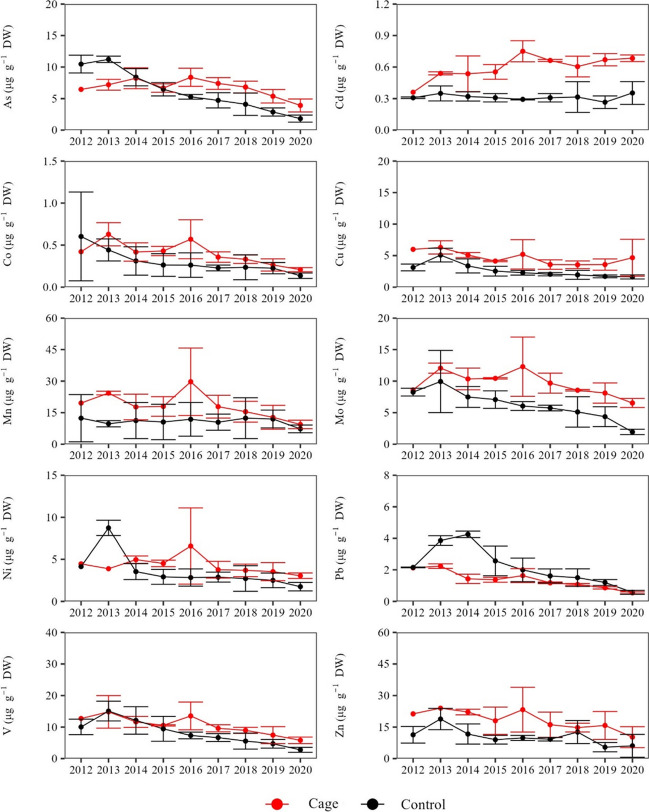
Mean (± SD) concentrations of TEs (μg g^−1^ DW) at each station in Site 1 during the period 2012–2020

**Fig. 5:**
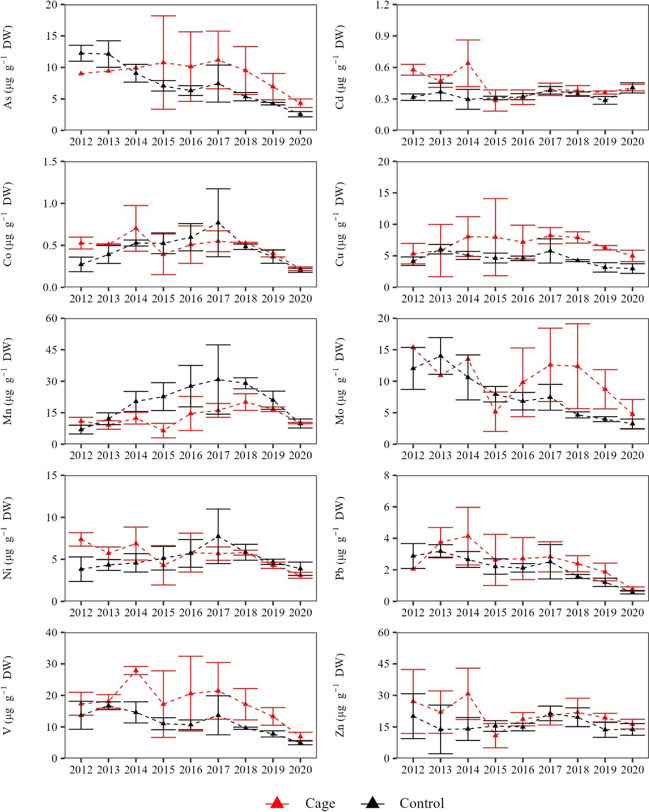
Mean (± SD) concentrations of TEs (μg g^−1^ DW) at each station in Site 2 during the period 2012–2020

TE concentrations in the dead sheaths for the period 2012–2020 (Table S2) showed a significant differentiation between the two stations in most cases (Fig. 4, Fig. 5, Table 1). Specifically, higher concentrations at the ‘Cage’ compared to the ‘Control’ stations were found for six out of ten TEs (As, Cd, Cu, Mo, V, Zn) in both sites. Additionally, in Site 1, higher concentrations of Co, Mn and Ni were also found at the ‘Cage’ station.

**Fig. 6:**
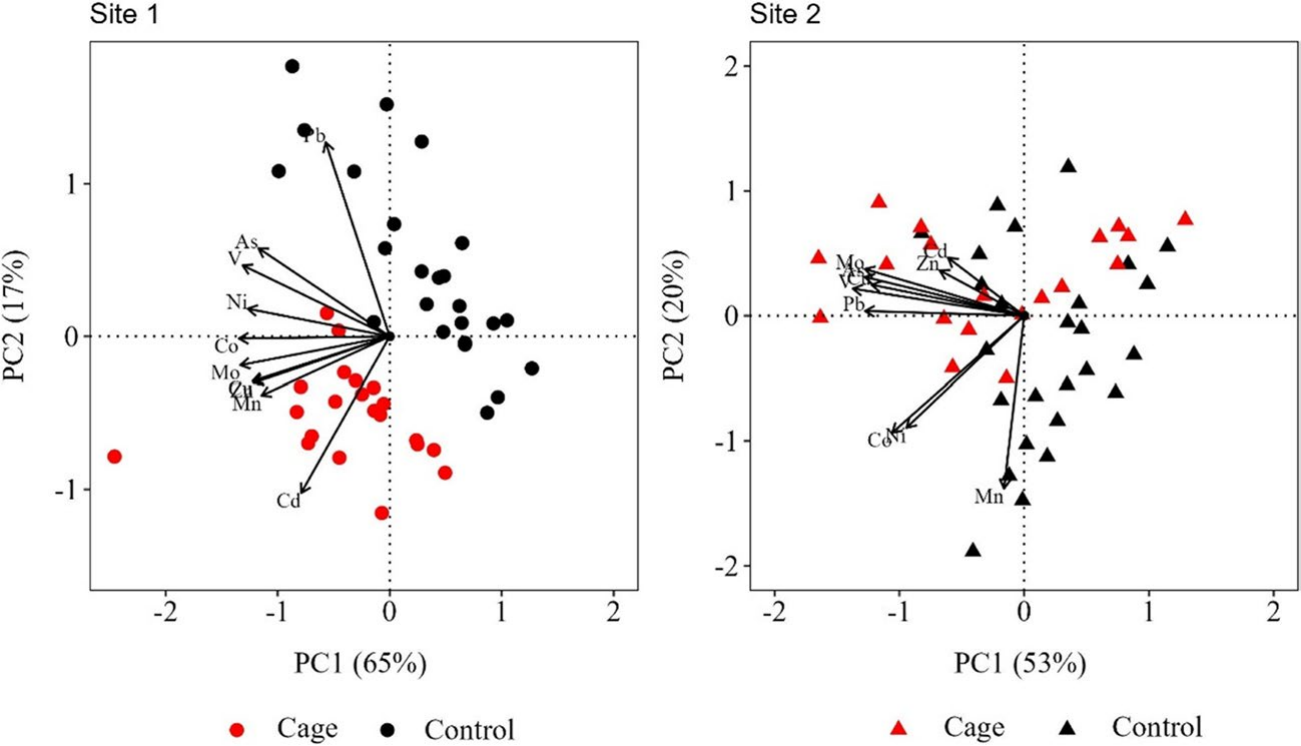
Principal component analysis (PCA) based on the Euclidean distances of TE concentrations of *P*. *oceanica* dead sheaths over the period 2012–2020 at each site and station

Overall, a prevailing decreasing trend was found in the concentrations of most TEs during the reconstructed period (Fig. 4, Fig. 5, Table 1). Two principal components (PC) explained the 82% and 73% of the variability of the TE concentrations in the dead sheaths along the 2012–2020 period in Site 1 and Site 2, respectively, with the PERMANOVA analysis indicating a significant separation of the two stations in each site (*p* < 0.05) (Fig. 6).

**Fig. 7:**
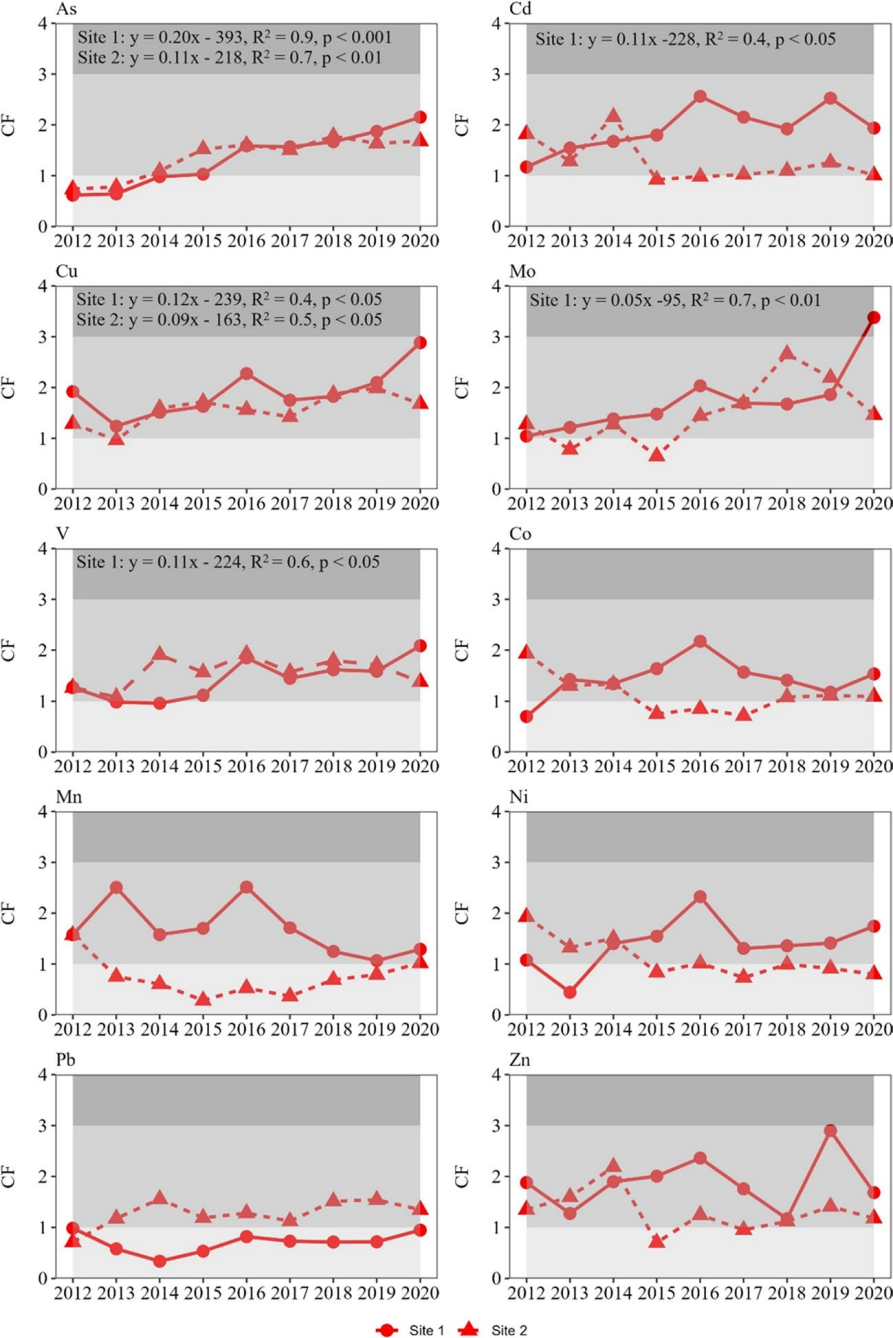
Trajectory of contamination factor (CF) of TEs over the period 2012–2020. In case of significant linear trends (*p* < 0.05), the corresponding linear regressions are also shown. Different shaded areas represent the three contamination level categories according to Hakanson 1980 (see ‘Materials and methods’ section)

Contamination levels in the proximity of the fish farm cages ranged from low to moderate and showed interannual fluctuations for all investigated TEs (Fig. 7). The CF was consistently moderate for most TEs in Site 1 and especially after the year 2014, while for Mo it was considerable in the year 2020. In Site 2, the CF was low throughout most of the investigated period. Some TEs exhibited an increasing contamination trend, namely As, Cd, Cu, Mo, V in Site 1 and, As and Cu in Site 2 (linear regressions, *p* < 0.05) (Fig. 7).

## Discussion

Our data analysis revealed overall higher TE concentrations and reduced rhizome production in the meadows adjacent to fish farm facilities. Throughout the 2012–2020 period, Site 1 exhibited predominately moderate contamination levels, whereas Site 2 showcased mostly low contamination levels. Nevertheless, both sites displayed an escalating trajectory of contamination in specific potentially toxic TEs which indicates a potential risk to nearby seagrass meadows.

Epiphytes close to the fish cages had high concentrations for most TEs, reaffirming their potential as valuable bioindicators (Sanz-Lázaro et al. 2011, 2012). It is noteworthy that despite their ecological importance, and their role as a substantial food source for herbivores (Tomas et al. 2005; Marco-Méndez et al. 2015) and thus as a mediator for element transfer into the higher trophic levels, they have been rarely assessed in relevant studies before (e.g., Sanz-Lázaro et al. 2012; Schlacher-Hoenlinger and Schlacher 1998). The increase of TEs in other compartments, particularly leaf blades, sheaths and rhizomes, in proximity to fish farm cages, aligns with the preferential accumulation of TEs observed in specific compartments (Malea et al. 2019a; Sanz-Lázaro et al. 2012) and is associated with the two primary uptake routes (water-to-leaf and sediment-to-root). The TE concentrations in the living compartments of *P*. *oceanica* shoots were similar or lower to the ones measured in previous studies. Specifically, the concentrations of Cu and Zn in the present study were similar to those measured in the rhizomes of *P*. *oceanica* close to fish farm cages, at 0 and 80 m (4.5 ± 0.5 and 2.8 ± 3 μg g^−1^ DW for Cu and 90 ± 13 and 52 ± 18 μg g^−1^ DW for Zn, respectively) (Pergent et al. 1999). However, the concentrations of TEs near the fish farm cages were lower than those in areas classified as polluted, such as the Gulf of Antikyra in Greece, where the TE concentrations in the leaf blades of *P*. *oceanica*, reached much higher concentrations than those reported here (Cu: 148 μg g^−1^ DW, Zn: 98 μg g^−1^ DW, Cd: 44 μg g^−1^ DW and Pb: 223 μg g^−1^ DW) (Malea et al. 1994). TE concentrations measured in the present study were also lower than those reported in the leaf blades of *P*. *oceanica* in proximity to mining activities, chemical plants and harbors (Co: 55.33 ± 14.19 μg g^−1^ DW, Ni: 1325 ± 5 μg g^−1^ DW, Pb: 44. 5 ± 4.5 μg g^−1^ DW) (Lafabrie et al. 2007b).

A clear differentiation of stations occurred, mostly in Site 1, showing enrichment of seagrass in TEs which was primarily driven by specific TEs such as Cd, Cu, Mo and Zn. These TEs have been previously identified as direct (e.g., Cu, Zn) or indirect (e.g., Cd, Mo) effluents of fish farm activities (Hamoutene et al. 2021) and their release has been associated with wastes originating from uneaten feeds and fish excreta (Grigorakis and Rigos 2011; Kalantzi et al. 2021) or with the use of antifouling paints on fish cage nets, like in the case of Cu and Zn (Nikolaou et al. 2014). Cd is usually found as an ingredient in fish feeds (Kalantzi et al. 2013) and is a non-essential heavy metal which is considered one of the most toxic for aquatic organisms (Kennish 2000). Previous studies have pointed out the moderate to considerable ecological risks associated with its release (Kalantzi et al. 2021), while its phytotoxic effects have also been previously described for other seagrass species, affecting the growth of seagrass *Cymodocea nodosa* (Malea et al. 2013) or *Zostera japonica* (Lin et al. 2016). Notably, this study presents new information on the concentrations of two TEs, Mo and V, in different compartments of *P*. *oceanica.* Both TEs are heavy metals that are found as part of fish feed ingredients but have received limited attention in previous studies (Copat et al. 2012; Sanz-Lázaro et al. 2012; Öztürk et al. 2012; Luy et al. 2012). This information is crucial due to the association of Mo with fish farm activities, as evidenced by a recent study on sediments surrounding many fish farms which indicated an enrichment of Mo in the sediments beneath the fish cages (Kalantzi et al. 2021). In addition, V has recently emerged as an important contaminant related to aquaculture activities (Ratcliff et al. 2016). Increased concentrations of these metals can inhibit plant growth by disrupting their metabolic pathways and their nutrient uptake, as has been described in the case of terrestrial plants (Aihemaiti et al. 2020).

The contamination levels during the 2012–2020 period were characterized as moderate in most cases, while an increasing trend of contamination was identified for Cd, Mo and V in Site 1 and As and Cu in both sites. The increasing tendency of contamination of these TEs is concerning, given the fact that they can be potentially phytotoxic (Zheng et al. 2018) and hamper seagrass performance (Boudouresque et al. 2020; Li et al. 2023).The increase in contamination could result in high concentrations of these TEs that have already been associated with leaf necrosis, reduced seagrass growth (Llagostera et al. 2016), impairments in the cytoskeleton (Malea et al. 2013) and even seagrass declines (Espel et al. 2019). However, our findings were not consistent between sites and elements, which should be interpreted in light of the complex interaction of factors controlling the abundance of the bioavailable forms of the TEs (Eggleton and Thomas 2004). Local environmental and physicochemical conditions, including geomorphology, temperature, organic matter content and redox potential, play a critical role in determining the availability of TEs for uptake from the water column and sediments (Kalantzi et al. 2013; Hamoutene et al. 2021). These factors influence the formation of ionic forms that can either facilitate or hinder the uptake and mobility of TEs (Li et al. 2023). For instance, the hydrodynamics may result in lateral transport of released TEs away from the immediately affected zone, with some TEs being assimilated fast by organisms close to the fish cages and transferred to long distances (> 1000 m) (Penry-Williams et al. 2022). The lower trophic levels of the ultra-oligotrophic environment of the Eastern Mediterranean basin often possess a high ability to assimilate inorganic compounds, like TEs, rapidly from the surrounding water (Basaran et al. 2010). Consequently, some TEs may be less abundant in the vicinity of these activities, a phenomenon that has been previously demonstrated for macro-nutrients as well (Pitta et al. 2009). Additionally, several studies have shown that TEs are mostly concentrated directly beneath the fish cages, while they diminish away from them, becoming almost negligible at short distances ( beyond 25 m) from the cages depending on local water circulation (Kalantzi et al. 2013, 2021). Thus, it is plausible that some of the released TEs did not reach the *P*. *oceanica* meadows which were located approximately 60 m away from the fish farming facilities. Finally, TEs may accumulate in the sediments and remain immobile for extended periods (Di Leonardo et al. 2017). The sediments beneath *P*. *oceanica* meadows, in particular, are considered long-term natural filters of TEs (Serrano et al. 2011; Lafratta et al. 2019) that effectively trap and immobilize the TEs over long periods, thereby decreasing their bioavailability.

It should be noted that the declining trends in some TE concentrations over the reconstructed period are likely linked to the inherent limitations in the method of lepidochronology which are related to the correlation between the mass loss of dead sheaths during the decomposition process and the concentrations of certain TEs which tend to remain stable (Malea et al. 2019b). The retention of a significant portion of TE concentrations during the decomposition is likely linked to the preferential accumulation of TEs in specific cell types which are less affected by the decay. This process may lead to higher concentrations of TEs in the older sheaths compared to the more recent ones (less degraded). In addition, a certain amount of TEs continues to be absorbed post-mortem, contributing as well to higher concentration of certain TEs in the older sheaths (Lafabrie et al. 2007b; Malea et al. 2019b). However, temporal trends of TE concentrations during the decay process are not consistent across all the TEs that have been assessed so far (e.g., Cd does not follow this pattern) and could vary between sites (Roméo et al. 1995; Ancora et al. 2004; Gosselin et al. 2006). Investigating the behaviour of additional TEs across different sites could significantly enhance our understanding of the trends in TE concentrations during the sheath decay process.

The lower *P*. *oceanica* rhizome production close to the fish farm cages suggests degradation of *P*. *oceanica* meadows close to the fish farms which could be attributed to several factors. Excess of nutrients and organic loads are commonly encountered in the vicinity of fish farm activities due to their release though various pathways, including uneaten feeds or fish faeces and excretion. These inputs can stimulate the growth of epibionts, such as algae or other organisms, which reduce the light availability and compete for resources (Ruiz et al. 2001) while they attract herbivorous species, leading to increased herbivory (Ruíz et al. 2009; Rountos et al. 2012). Moreover, the excess of nutrients and organic loads can alter the physical and chemical characteristics of sediments and lead to hypoxic/anoxic conditions and subsequent sulphate reduction which can deteriorate seagrass performance (Holmer et al. 2008; Boudouresque et al. 2020; Howarth et al. 2022).

In sum, the low to moderate contamination levels in the vicinity of the fish cages coupled with the lower rhizome production observed near them show signs of impact stemming from the fish farming activities. It is important also to highlight the upward trend in the contamination levels of certain potentially toxic TEs (As, Cd, Cu, Mo, V). Therefore, it would be important to implement comprehensive monitoring of the water quality and sediment conditions around each fish farm, accompanied by the adoption of corrective measures, if necessary, that can include new innovative technologies, optimization of feed compositions and monitoring of the nutrient run off. Since TE release related to fish farms frequently exhibits notable dissimilarities across various locations (Eggleton and Thomas 2004; Kalantzi et al. 2021), as evidenced by the differences in our study sites as well, it is important that any monitoring strategy would account for the specific distinctive characteristics inherent to each ecosystem. These measures are essential to protect and preserve the local ecosystems while ensuring the production of high-quality products, especially in the context of the current UN Blue Transformation Strategy, which sets new goals towards a more ecologically aware and sustainable industry that will ensure not only improved production and nutrition, but also an environment with low environmental footprint.

### Supplementary Information

Below is the link to the electronic supplementary material.

Supplementary file1 (XLSX 28 KB)

Supplementary file2 (DOCX 151 KB)

## Data Availability

Data available upon request.
