# Supplementary material for: Trajectories of trace element accumulation in seagrass (Posidonia oceanica) over a decade reveal the footprint of fish farming
Source: Environ Sci Pollut Res Int. 2024 Mar 26;31(19):28139–52. doi: 10.1007/s11356-024-32910-0 (PMC11058863; doi:10.1007/s11356-024-32910-0)
Supplement: Supplementary file 2 — Supplementary file2 (DOCX 151 KB) [file 11356_2024_32910_MOESM2_ESM.docx]

**Trajectories of trace element accumulation in seagrass (*Posidonia oceanica*) over a decade reveal the footprint of fish farming**

Victoria Litsi-Mizan ^1,2^, Ioanna Kalantzi ^2^, Manolis Tsapakis ^2^, Spiros A. Pergantis ^3^, Ioannis Karakassis ^1^, Eugenia T. Apostolaki ^2 *^


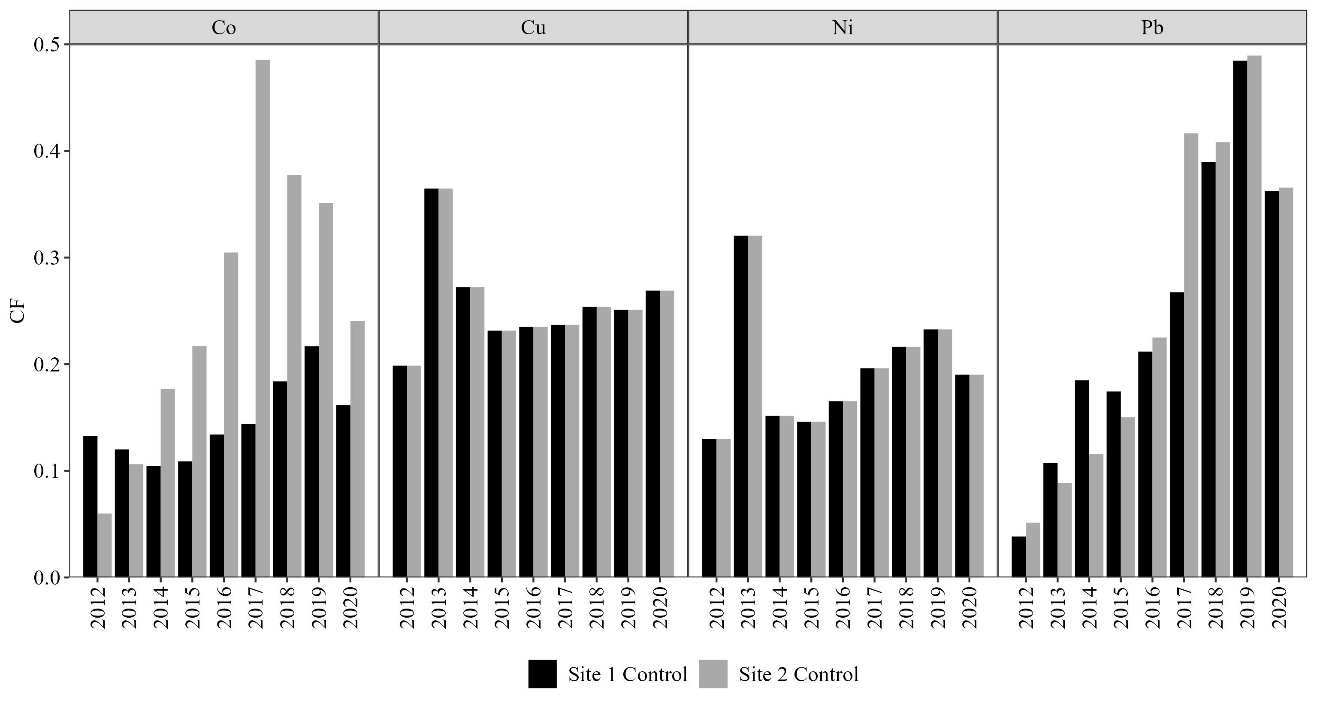


Fig. S1. Contamination levels of the selected ‘Control’ stations based on the contamination factor (CF) calculated by applying the MaLE method (Malea et al. 2019b). The levels of contamination were characterized as follows: CF < 1: low contamination; CF < 3: moderate contamination, CF < 6: considerable contamination, CF.6: very high contamination.
